# Clinical outcomes in alpha-mannosidosis: a systematic review of therapeutic approaches

**DOI:** 10.1186/s13023-026-04394-3

**Published:** 2026-07-22

**Authors:** Arezki Azzi, Reem Bin Shlhoob, Hassan Al-Shehri

**Affiliations:** 1 Department of Biochemistry, College of Medicine, Imam Mohammad Ibn Saud Islamic University (IMSIU), P.O. Box 7544, Riyadh, 4233-13317 Saudi Arabia; 2 College of Medicine, Imam Mohammad Ibn Saud Islamic University (IMSIU), P.O. Box 7544, Riyadh, 4233-13317 Saudi Arabia; 3 Department of Pediatrics, College of Medicine, Imam Mohammad Ibn Saud Islamic University, P.O. Box 7544, Riyadh, 4233-13317 Saudi Arabia

**Keywords:** Alpha-mannosidosis, Hematopoietic stem cell transplantation, Enzyme replacement therapy, Lysosomal storage disorder, Current treatments

## Abstract

**Background:**

Alpha mannosidosis (AM) is a rare lysosomal storage disorder caused by a deficiency in the α-mannosidase enzyme, resulting in impaired glycoprotein metabolism within lysosomes. Enzyme dysfunction is attributed to an autosomal recessive mutation in the MAN2B1 gene. Affected individuals present with a broad spectrum of manifestations, including developmental delays, cognitive decline, musculoskeletal abnormalities, hearing difficulties, and recurrent infections. Current therapeutic options are limited to hematopoietic stem cell transplantation and the more recently developed enzyme replacement therapy.

**Objective:**

The aim of this review was to evaluate and compare the therapeutic outcomes, benefits and challenges associated with Hematopoietic stem cell transplantation (HSCT) and enzyme replacement therapy (ERT) in the treatment of AM.

**Methods:**

A systematic search across PubMed, MEDLINE, EMBASE, the Cochrane Library, OMIM, and ScienceDirect identified 12 original studies from 307 records. The data are presented narratively due to the scarcity of literature and the heterogeneity of study designs and interventions.

**Results:**

A total of 28 patients who received hematopoietic stem cell transplantation showed improvements in preserving neurocognitive function and skeletal stabilization and reduced infection rates, especially when performed at relatively young ages. However, this treatment carries significant risks, including infections, graft-versus-host disease, and increased morbidity and mortality, particularly in older patients. Conversely, enzyme replacement therapy was administered to 75 patients, who demonstrated a favorable safety profile, enhanced respiratory function, reduced skeletal abnormalities, and improved overall quality of life. However, enzyme replacement therapy has limited efficacy in preventing neurocognitive decline and requires lifelong administration.

**Conclusion:**

Both interventions yield better outcomes when initiated early, particularly before cognitive deterioration becomes significant. This review emphasizes the importance of a timely diagnosis to optimize treatment outcomes and prevent severe complications.

## Introduction

Alpha-mannosidosis (AM) (OMIM 248500) is an ultrarare lysosomal storage disorder that occurs in approximately 1 in 500,000–1,000,000 live births [1]. It is caused by an autosomal recessive genetic mutation in the MAN2B1 gene on chromosome 19 (19p13.2-q12). This mutation results in a deficiency of lysosomal alpha-mannosidase, an enzyme essential for breaking down glycoproteins within lysosomes. Consequently, inadequate enzyme activity leads to the buildup of undigested material within lysosomes, disrupting cellular processes [2, 3].

Affected individuals exhibit variable ages of onset, progression, and severity [4]. Initially, most individuals appear normal at birth but later develop progressive symptoms [5]. AM is characterized by nonspecific, multisystemic manifestations, including sensorineural hearing loss and frequent infections due to an underlying immune deficiency. Developmental delays are frequent, along with low muscle tone, ataxia, and spastic paraplegia. Musculoskeletal abnormalities such as scoliosis, deformities of the sternum, and signs of dysostosis multiplex, along with radiological findings such as dysostosis multiplex, focal sclerotic lesions, osteonecrosis, and osteopenia, are often observed, in addition to joint problems and muscular weakness. The distinct facial features include a large head, prominent forehead, rounded eyebrows, a flattened nasal bridge, an enlarged tongue, widely spaced teeth, and a protruding lower jaw. Psychiatric symptoms, including episodes of psychosis, may develop over time. Rheumatologic issues, especially systemic lupus erythematosus, are also observed. Gastrointestinal complications such as chronic diarrhea, difficulty swallowing, aspiration, and enlargement of the liver and spleen are common. Other complications can include poor growth; eye abnormalities, such as retinal degeneration, mild strabismus, and optic nerve atrophy; adult-onset cardiac issues; and lung involvement, such as interstitial lung disease [5, 6].

Notably, there is no correlation between genotype and phenotype, reflecting disease heterogeneity [7]. Recently, three subtypes have been defined: Type 1, characterized by a milder form of the disease that presents clinically after the age of 10 years, does not involve skeletal abnormalities and progresses slowly; Type 2, presenting as a moderate form, is clinically identifiable before the age of 10 years, is accompanied by skeletal abnormalities, and progresses slowly with the onset of ataxia typically in the second or third decade of life; and Type 3, representing the severe form, is recognizable in infants, featuring skeletal abnormalities and clearly exhibiting progression, leading to early mortality owing to primary central nervous system involvement or myopathy [8]. As a result of insidious neuromuscular and skeletal decline, most patients eventually become wheelchair dependent and struggle to maintain their social independence.

Given the nonspecific presentations that can overlap with other lysosomal storage disorders, making a clinical diagnosis is challenging and is primarily useful for raising suspicion rather than confirming the disease. A family history of consanguinity or similar clinical features in relatives can further support the likelihood of disease inheritance [5]. Preliminary diagnostic features include the presence of vacuolated lymphocytes in peripheral blood or bone marrow smears and increased levels of mannose-rich oligosaccharides in the urine, which can be identified through techniques such as thin-layer chromatography or capillary high-performance anion exchange chromatography. While these tests are useful for screening, they are only suggestive of AM. A conclusive diagnosis requires measuring acidic alpha-mannosidase activity in leukocytes or other nucleated cells, such as fibroblasts [9]. Genetic testing provides further confirmation, with approaches such as single-gene analysis, lysosomal multigene panels, or comprehensive sequencing, which offer a diagnostic yield of approximately 98.5% [6, 10].

The management of AM primarily involves symptomatic treatment and the prevention of complications. Hematopoietic stem cell transplantation (HSCT) is the oldest treatment modality [11] and is recommended for preserving neurocognitive function [11–18] and addressing other symptoms. However, it carries significant risks of morbidity and mortality [11–13, 17], despite newer protocols aiming to increase its safety [16, 18]. A newer treatment is enzyme replacement therapy (ERT) with recombinant human velmanase alfa (VA), which is a recombinant enzyme that reaches lysosomes through mannose-6-phosphate receptors and breaks down accumulated mannose-rich oligosaccharides [19]. Clinical trials have confirmed its safety [20–23], although limitations exist, including the lack of evidence for the ability of the recombinant enzyme to cross the blood‒brain barrier and improve cognitive function [24].

Given the rarity of AM, the literature on the effectiveness and indications of therapeutic options is limited. Hence, the aim of this systematic review was to evaluate the efficacy and toxicity of current therapeutic interventions for AM, providing the latest information to inform clinical practice on disease treatment options.

## Methods

### Search strategy

Considering the scarcity of literature, multiple databases were used to identify relevant studies, including PubMed, EMBASE, the Cochrane Central Register of Controlled Trials (CENTRAL), OMIM, and ScienceDirect, following preferred reporting items for systematic reviews and meta-analyses (PRISMA) guidelines [25]. The search term used in PubMed was “alpha-mannosidosis” with the medical subject heading (MeSH) terms AND treatment OR therapy OR therapeutic intervention. In the other databases, the terms alpha mannosidosis AND treatment OR therapy OR therapeutic intervention were used. We included studies published in English, and for evidence of the efficacy and effectiveness of the treatments, we included randomized controlled trials (RCTs), cohort studies, and before-and-after studies. For evidence of toxicity, we included RCTs, cohort studies, before-and-after studies, and case reports, which are weaker forms of evidence. The systematic literature search was performed up to June 2024.

### Study selection and screening

**Fig. 1:**
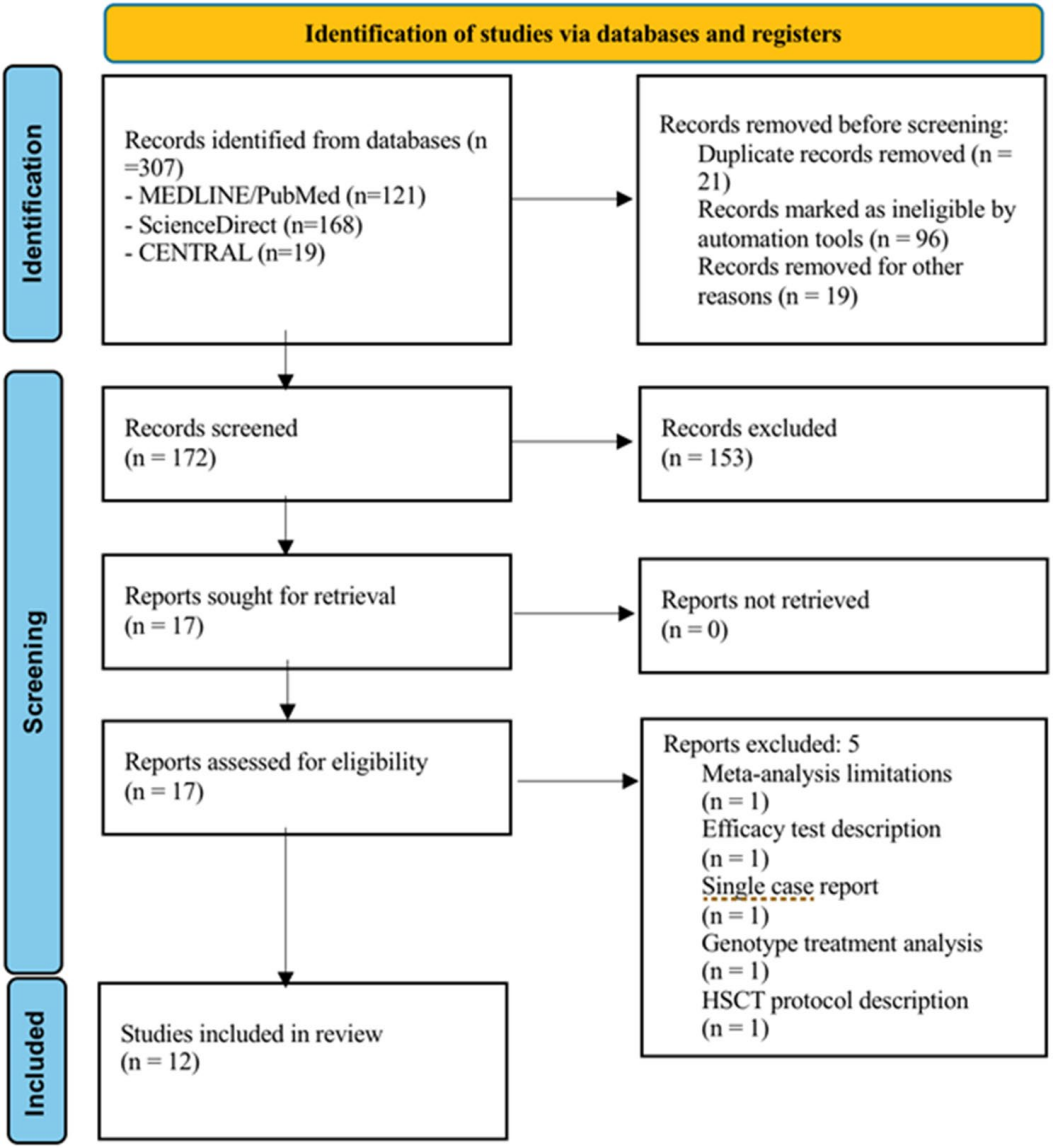
Flow diagram for evaluating the efficacy and safety of therapeutic interventions for alpha-mannosidosis (AM)

All the included studies were screened using Rayyan [26] by two independent reviewers (R.A. and S.A.) to ensure consistency in the selection of studies. Duplicate studies, in vivo studies, and animal model studies were excluded after title screening. There were no discrepancies, as both reviewers yielded the same results. The two independent reviewers then screened the abstracts, excluding studies that were irrelevant to therapeutic interventions of AM. After the full texts were retrieved, some studies were subsequently excluded because of their irrelevance to the study’s objective, as illustrated in detail in Fig. 1.

### Data extraction

Two reviewers independently extracted data from the eligible studies. In cases of discrepancies, the opinion of a third reviewer was sought to reach a consensus. The extracted data were then entered into a standardized spreadsheet (Table 1). The following information was selected: (1) author, year, country, study scope, study design, sample size, intervention, age at diagnosis (mean), age at start of intervention (mean), treatment duration in months (mean), follow-up duration in months, and quality score (number/total). (2) Outcomes focusing on relevant AM manifestations. Heterogeneity in study design and intervention outcomes, as well as the limited number of studies, prevents a meta-analysis of the data.

**Table 1:** Characteristics of the included studies

Author, year	Country	Study scope	Study design	Sample size	Intervention	Age at diagnostic (mean)	Age at start of intervention (mean)	Treatment duration, months (mean)	Follow-up, months (mean)	Quality score (no./total)
Guffon et al. [20], 2022	MC	MC	Longitidual clinical trial	5	1 mg/kg of VA IV once a week	NR	4.5	27.5	16	6//9
Lund et al. [21],2018	MC	MC	Longitidual clinical trial	33	1 mg/kg of VA IV once a week	NR	NR	29.3	48	5//9
Borgwardt et al. [22],2013	Denmark	SC	RCT	10	25 U/kg or 50 U/kg of VA	NR	12.6	12	12	4//5
Grewal et al. [12],2004	USA	SC	Case series	4	HSCT, unrelated T-cell–depleted donor grafts as follows: patients 1 and 2, HLA-matched marrow; patient 3, HLA 1-antigen mismatched marrow; and patient 4, HLA 2-antigen mis- matched cord blood.	7.8	9.25	NA	72	8//12
Borgwardt et al. [23], 2018	Denmark	MC	RCT	25	1 mg/kg of VA IV once a week		NR	13	12-18 for placebo-group 24-36 months for active treatment-group	4//5
Mynarek et al. [13], 2012	New Zealand	MC	Cohort study	17	Allogeneic HSCT, first transplants were performed from a matched related donor in four cases (of which two were an HLA-identical sibling, one an HLA-identical mother and one an HLA-identical other family member), matched unrelated BM or PBSC donor in five cases, a mismatched unrelated donor in two cases and a haplo identical family donor in two cases. Four patients received an unrelated cord blood graft: two of those with 4/6 HLA-matches, one with 5/6 HLA-match and one classified as ‘matched’.	2.5	3.6	NA	66	6//9
Yesilipek et al. [14], 2012	Turkey	SC	Case series	2	HSCT, patient A HLA-C allele-mismatched, unrelated donor, Patient B 10/10-matched, unrelated donor	7	9.5	NA	Patient A: 24 months patient B: 8 months	8//12
Wall et al. [15], 1998	USA	SC	Case report	1	Allogenic HSCT, HLA- identical brother as donor.	19 months	22 months	NA	19	11//12
Will et al. [11], 1987	UK	SC	Case report	1	Allogenic HSCT, one haplotype mismatch but same HLA DR	7	7	NA	3	11//12
Albert et al. [16], 2003	Germany	SC	Case report	1	PBSCT, HLA phenotypically identical mother.	23 months	2	NA	27	11//12
Broomfield et al. [17], 2010	UK	SC	Case series	2	HSCT, Patient A HLA-matched family member, Patient B HLA-matched father donor	Patient A: 3 years Patient B: asymptomatic	Patient A: 13 years Patient B: 6 months	NA	Patient A: 36 Patient B: 72	11//12
Santoro et al. [18], 2023	Italy	SC	Case report	1	HSCT+ERT, from a 6/8 matched unrelated cord blood unit	5	ERT: 7 months HSCT: 9 months	ERT: till 3 months after HSCT	36	11//12

MC Multicenter, SC Single center, RCT Randomized clinical trials, VA Valmenase Alfa, HSCT Hematopoietic stem cell transplantation, HLA Human leukocyte antigen, PBSCT Peripheral blood stem cell therapy, ERT Enzyme replacement transplantation, NR Not reported, NA not applicable

### Quality assessment

Quality assessment was performed using the Newcastle‒Ottawa scale for longitudinal clinical trials [27], the Jadad scale for RCTs [28], the Joanna Briggs Institute scale for case series [29], and the case report scale for case reports [30].

## Results

### Study selection

The initial search identified 307 records. Among these, 136 were excluded on the basis of their titles by automated tools and two independent reviewers, whereas an additional 153 were removed after the abstracts were reviewed. The full texts of the remaining 17 records were retrieved and evaluated for eligibility. Five studies were removed because they reported the natural history of AM, analyzed genotypes, described efficacy test protocols, or outlined HSCT engraftment protocols, as shown in Fig. 1.

### Study characteristics

Six studies were conducted in Denmark, the USA, and the UK, with two studies from each country. Four additional studies took place in New Zealand, Turkey, Germany, and Italy, along with two multicentric studies. The publication dates span from 1987 to 2023. An overview of the characteristics of the 12 included studies is provided in Table 1.

Studies reporting on HSCT shared similarities in sample size and outcome measures but differed in certain transplantation protocols. Four studies reported single cases, while two case series each included two patients, another case series investigated four patients, and a cohort study included 17 patients, resulting in a total of 28 patients. The mean age at diagnosis was 3.8 years, with a range from 7.8 to 0.4 years. At the start of transplantation, the age ranged from 0.8 to 13 years, with an average of 5 years. Given the permanence of the intervention, the duration of treatment is not applicable. The mean follow-up period was 36 months, ranging from 3 to 72 months. The quality of the included studies was assessed as moderate to high on the basis of the assessment scales.

The studies evaluating ERT were similar in terms of sample size, intervention, and outcome measures. Two RCTs and two other longitudinal studies were included. The sample sizes ranged from 5 to 33, totaling 75 participants. The ages of the participants at diagnosis were not reported, whereas the average age of intervention was 8.55 years, ranging from 4.5 to 12.6 years, as described in two studies. The mean duration of therapy was 20.5 months, with a range of 12–29.3 months. Within a 12–30 month range, the average follow-up duration was 26.5 months. The quality of the included studies was rated as moderate to high.

### Clinical outcomes

#### Hematopoietic stem cell transplantation

The efficacy and toxicity of HSCT in patients with AM were evaluated qualitatively through eight clinical outcomes on the basis of biomarkers of the disease, as documented in eight studies (Table 2). Cognitive function and musculoskeletal defects were the most frequently reported outcomes, highlighting the positive effect of HSCT (7/8) [12–16, 18], followed by changes in oligosaccharide levels, which also showed beneficial results (6/8) [13–16, 18]. The reported effects on hearing impairment [12–14, 16] and central nervous system (CNS) involvement [13, 16–18] were diverse, ranging from improvement to no change or deterioration (4/8). Improvements in respiratory function were the least reported outcome (2/8) [15, 17]. In terms of mortality, four deaths following HSCT were recorded among 22 patients across three studies (3/8) [11, 13]. The other two studies reported contrasting results, indicating that HSCT was safe with no deaths or long-term consequences (2/8) [16, 18].

**Table 2:** Summary of findings for alpha-mannosidosis (AM) patients receiving hematopoietic stem cell transplantation (HSCT)

Outcomes	Number of articles	Number of AM patients with the outcome described	Main findings	References
Mortality	3	22	All patients had acute graft-versus-host disease GVHD, and one patient additionally developed chronic GVHD. Four patients experienced severe sepsis requiring ventilatory support, resulting in two deaths. Acute GVHD (grade II or higher) occurred in two patients, one of whom died, while chronic GVHD affected six patients, involving the skin; all patients achieved remission by the last follow-up. Bronchiolitis obliterans was observed in two patients. One patient died due to bronchopneumonia after 18 weeks post-transplantation.	Grewal et al. [12] Mynarek et al. [13] Will et al. [11]
Safety	3	4	No signs of chronic GVHD or other long-term side effects related to the transplant. Patient A had a brief episode of GVHD and multiple recurrent pulmonary infections.	Albert et al. [16]; Santoro et al. [18] Broomfield et al. [17]
Cognitive function	7	28	HSCT is linked to improvements in both functional and social skills, and it can halt the progressive neurocognitive decline while potentially enhancing intellectual function. Pediatric patients stayed socially active despite needing special education. One of the two adult patients lived independently. Patient A’s intelligence score decreased from 52 at the time of transplantation to 48. Patient B, being younger, showed better improvement in adaptive skills and had milder clinical deficits. Patient A showed improvement in expressive speech. Patient B had good expressive language and was attending regular school.	Grewal et al. [12]; Wall et al. [15]; Albert. et al. [16]; Santoro et al. [18] Mynarek et al. [13] Yesilipek et al. [14] Broomfield et al. [17]
Hearing impairment	4	24	Hearing of speech frequencies (500–3000 Hz) showed potential for improvement after HSCT. Improved communication skills were observed following HSCT, although hearing function remained the same. Significant improvements in both language skills and hearing ability were noted following PBSCT. Among 13 patients, hearing aids were discontinued in three due to improved hearing. One patient developed progressive hearing loss requiring aids five years post-HSCT, and three others needed hearing aids after initially not requiring them pretransplant. Only one patient retained normal hearing.	Grewal et al. [12] Yesilipek et al. [14] Albert et al. [16] Mynarek et al. [13]
Muscloskeletal defects	7	28	Following HSCT, there was a noticeable improvement in coarse facial features, with significant enhancements in bony abnormalities, particularly in the skull, thoracolumbar spine, and metacarpals. The trabeculation of the long and short bones returned to normal, and there were no joint limitations observed. Out of 15 patients, three showed no progression of dysostosis multiplex, while four experienced ongoing issues, including surgeries for hip subluxation, knee arthroplasty, and pes equinus correction. Patient A experienced a deterioration of scoliosis, while Patient B exhibited a slight genu valgum and mild hyperreflexia in the lower limb deep tendon reflexes.	Grewal et al. [12]; Yesilipek et al. [14]; Wall et al. [15]; Albert et al. [16]; Santoro et al. [18] Mynarek et al. [13] Broomfield et al. [17]
CNS involvement	4	21	Post-HSCT cerebral MRI results indicated nonspecific white matter changes in three patients, reduced white matter volume in one, and decreased myelination in two, with one case normalizing. Arnold–Chiari malformation was identified in three patients. Delayed myelination observed before HSCT normalized after 12 months. There were no significant changes in T2 hyperintensity or cerebrospinal fluid spaces since diagnosis. Patient A exhibited increased cerebral volume loss, particularly in the cerebellum, while Patient B had normal cranial MRI but showed spinal abnormalities.	Mynarek et al. [13] Albert et al. [16] Santoro et al. [18] Broomfield et al. [17]
Respiratory function	2	3	No lung infections or signs of reactive airway disease were found. Patient A did not exhibit any further respiratory flare-ups, while Patient B experienced mild pectus carinatum	Wall et al. [15] Broomfield et al. [17]
Oligosaccharide levels (serum/urine)	6	23	Mannosidase activity was within normal ranges after transplant, and oligosaccharide levels in the serum and urine decreased. The spleen and liver had normal levels of acid α-mannosidase activity, whereas the brain only demonstrated 7% activity.	Mynarek et al. [13]; Yesilipek et al. [14]; Wall et al. [15]; Albert et al. [16]; Santoro et al. [18] Will et al. [11]

GVHD graft-versus-host disease, PBSCT Peripheral blood stem cell transplantation

##### Safety and mortality

Among the four patients who received unrelated T-cell-depleted donor grafts, all developed acute graft-versus-host disease (GVHD), and one patient also developed chronic GVHD [12]. Another patient who received allogenic HSCT with a haplotype mismatch but matched HLA DR died due to pulmonary complications 18 weeks posttransplantation [11]. Additionally, two patients died due to severe sepsis requiring ventilatory support, and another died from acute GVHD (grade II or higher) [13]. Notably, modified transplantation protocols resulted in safer outcomes. No signs of chronic GVHD or other long-term transplant-related side effects were observed in patients who had received T-cell-depleted peripheral blood stem cell transplantation (PBSCT) or ERT following transplantation [16, 18].

##### Cognitive function

While the extent of improvement varied [14–17], pediatric HSCT recipients generally achieved better outcomes than adult HSCT recipients [13–14]. Although they required special education, most pediatric HSCT recipients showed social and functional improvements [12, 15, 16, 18]. Mynarek et al. reported that, of the two adult HSCT recipients in their cohort, one was able to live independently [13]. The impact of HSCT on neurocognitive function in relation to age at the time of transplant was clearly demonstrated by Yesilipek et al. [14]. Older patients exhibited a decline in intelligence scores posttransplant, whereas younger patients exhibited improved adaptive skills and milder clinical deficits posttransplant.

##### Hearing impairment

The improvement in hearing loss varied, with one study highlighting enhancements in hearing ability and subsequent language skills following PBSCT [16], whereas others reported improvements only in language skills following allogenic HSCT [14]. Additionally, some studies highlighted potential improvements in hearing speech frequencies in the range of 500–3000 Hz [12], whereas four out of 13 participants experienced deterioration in hearing function [13].

##### CNS involvement

Other structural neurological changes varied according to patient age and the method of transplantation. A positive neurological effect observed was the normalization of delayed myelination following PBSCT [16]. Administering ERT before HSCT did not result in changes in cerebral magnetic resonance imaging (MRI) or cerebrospinal fluid (CSF) spaces [18]. Notably, undergoing HSCT at a later age may be associated with more severe cerebral changes, as evidenced by increased cerebral volume loss, particularly in the cerebellum [17].

##### Musculoskeletal defects

Partial alleviation of bony deformities and coarse facial features was observed following transplantation, with no joint abnormalities [12, 14–16, 18]. However, not all patients experienced these benefits; four patients continued to have issues and required surgical intervention for their bony conditions [13]. Moreover, one patient who underwent HSCT at the age of 13 years experienced worsening of scoliosis [17].

##### Respiratory function

In both studies where respiratory outcomes were reported [15, 17], all patients experienced respiratory improvement and resolution of recurrent lung infections. Only one patient developed mild pectus carinatum [17].

##### Oligosaccharide levels (serum/urine)

Following transplantation, enzyme activity levels returned to normal, and oligosaccharide levels in both serum and urine decreased [13–16, 18].

#### Enzyme replacement therapy

Four studies investigated the effectiveness and side effects of ERT through nine clinical outcomes (Table 3). Cognitive function was evaluated via various tools [20, 22]; although the results were not significant, improvements over the initial baseline were observed (2/4). Hearing function remained stable following therapy (2/4) [20, 22]. The impact on musculoskeletal defects was positive, with notable differences in the static significance of motor subtests (4/4) [20–23]. CNS biomarkers showed a positive shift in one study, whereas another reported no change (2/4) [21, 22]. Immune dysfunction was positively reversed, as indicated by increased serum immunoglobulin G (Ig) levels (4/4) [20–23], whereas one study noted variations in other Ig serum levels [20]. Positive effects on respiratory function (3/4) [21–23], quality of life (QoL) (1/4) [20], and decreased oligosaccharide levels (4/4) [20–23] have been reported. There were no reported fatalities, and despite some adverse effects, the safety profile was considered acceptable in all studies (4/4) [20–23].

**Table 3:** Summary of findings for alpha-mannosidosis (AM) patients receiving enzyme replacement therapy (ERT)

Mortality			No mortality was reported	
Safety	4	73	The safety profile was acceptable, demonstrating good tolerance in all children and suitability for long-term use.	Guffon et al. [20]; Lund et al. [21]; Borgwardt et al. [22,23]
Cognitive function	2	15	Although improvements in age-equivalent and raw MSEL scores varied among children, most patients showed enhancements. Patients with initially lower total equivalence ages compared to their chronological ages demonstrated a mean increase of approximately 4 months, with insignificant changes observed in Leiter subtests.	Guffon et al. [20] Borgwardt et al. [22]
Hearing impairment	2	15	There was no worsening of hearing impairment observed. Bone conduction thresholds remained stable at median and minimum levels but varied more at maximum thresholds, with improved hearing at 500 Hz and decreased hearing at 2000 Hz.	Guffon et al. [20] Borgwardt et al. [22]
Muscloskeletal defects	4	73	Pediatric patients demonstrated greater improvement in both the 3MSCT and 6MWT compared to adults, with those having the longest treatment exposure showing a mean absolute increase at the end of the follow-up period. While mean values increased for both the 3MSCT and 6MWT, only the increase in the 3MSCT was statistically significant. Most BOT-2 subtests, except for the balance subtest, showed statistically significant improvements. Pediatric patients receiving VA had better 3MSCT results compared to adults, although the difference was not statistically significant.	Guffon et al. [20]; Lund et al. [21] Borgwardt et al. [22] Borgwardt et al. [23]
CNS involvement	2	43	No significant changes in CSF biomarkers were observed from baseline. CSF-oligosaccharides exhibited a gradual and continuous decline.	Lund et al. [21] Borgwardt et al. [22]
Immune dysfunction	3	63	A notable increase in serum IgG levels was observed. IgA levels increased, whereas IgM concentrations declined among the children.	Guffon et al. [20]; Lund et al. [21]; Borgwardt et al. [23] Guffon et al. [20]
Respiratory function	3	68	The increase in FVC was more notable in pediatric patients compared to adults.	Lund et al. [21]; Borgwardt et al. [22,23]
Oligosaccharide levels (serum/urine)	4	73	Serum oligosaccharide levels decreased following treatment administration.	Guffon et al. [20]; Lund et al. [21]; Borgwardt et al. [22,23]
Quality of life	1	5	All children demonstrated improvement in all aspects, including self-care, mobility, and social function.	Guffon et al. [20]

MSEL Mullen Scales of Early Learning, 3MSCT 3-Minute Stair Climb Test, 6MWT 6-Minute Walk Test, BOT-2 Bruininks-Oseretsky test of motor proficiency, VA Velmanase alfa, CNS central nervous system, CSF cerebrospinal fluid, Ig immunoglobulin, FVC forced vital capacity

##### Safety and mortality

ERT demonstrated good tolerability in all the children studied, and while participants experienced various adverse reactions, no deaths were reported [20–23]. In a cohort composed of 10 patients receiving ERT, two had mild–moderate infusion-related reactions, and both developed rhLAMAN immunoglobulin G antibodies with no neutralizing effect [22].

##### Cognitive function

Cognitive function tests have yielded varying results among children, with most showing improvements from their baseline levels [20, 22]. Patients whose initial total equivalent ages were lower than their chronological ages showed an average increase of approximately 4 months [22]. Enhancements in the nonverbal intelligence and cognitive ability tests were not statistically significant in some cases [22].

##### Hearing impairment

Bone conduction thresholds varied significantly at maximum thresholds, with better hearing at 500 Hz and worse hearing at 2000 Hz [22]. At the median and minimum levels, the bone conduction thresholds were constant, and there was no worsening of hearing impairment [20, 22].

##### CNS involvement

No study has reported the effect of ERT on structural neurological defects, if present. However, among those that addressed related biomarkers, one study reported that baseline CSF biomarkers remained unchanged [21], whereas another reported a progressive and consistent decline in CSF oligosaccharides [22].

##### Musculoskeletal defects

All the motor subtests, apart from the balance test, revealed a mean increase by the end of the follow-up period when the patients received the longest duration of therapy [20–22]. Further analysis of fine motor accuracy, manual dexterity, upper limb coordination, and bilateral coordination revealed greater improvements in pediatric patients [36]. When the 3-minute stair climb test (3MSCT) and the 6-minute walk test (6MWT) were compared with those of adult patients, pediatric patients improved more often [20–21, 23].

##### Respiratory function

When respiratory function was assessed, the increase in forced vital capacity (FVC) was more pronounced in pediatric patients than in adults [21–23].

##### Oligosaccharide levels (serum/urine)

There were decreased levels of oligosaccharides in the serum and CSF [20–23].

##### Immunological dysfunction

The immune system responded positively, with considerable increases in blood IgG levels [20–21, 23], while some patients also presented elevated IgA levels. Conversely, children’s IgM concentrations declined [20].

##### Quality of life

Children’s quality of life gradually increased in several domains, including self-care, mobility, and social function [20].

## Discussion

In this systematic review, we focused on the therapeutic outcomes, benefits and challenges associated with HSCT and ERT for the treatment of AM.

The greater benefit observed in pediatric recipients underscores the importance of early intervention with HSCT to maximize its efficacy [13–17]. These improvements from early transplantation align with survey findings that HSCT recipients who underwent transplantation before 6 years of age had enhanced physical abilities such as coordination and independent walking. Moreover, they had improved speech and hearing and a better quality of life for both patients and their caregivers. These findings resulted in higher EQ-5D-5 L utility values than in those receiving only supportive care [31]. The timing of HSCT appears to play a major role, as evidenced by worsening neurological changes when patients underwent HSCT at more advanced ages [17].

Universal normalization of enzyme activity levels and a reduction in oligosaccharide levels in both serum and urine suggest that HSCT is effective for this parameter [13–16, 18]. However, the limited reporting of respiratory function improvement and the resolution of recurrent lung infections may indicate that, compared with other disease manifestations, HSCT is less effective in addressing respiratory symptoms [15, 17].

The outcomes following PBSCT were superior to those following traditional HSCT, particularly in terms of hearing ability and language development [16]. Improvements in delayed myelination were also more pronounced, suggesting that PBSCT may become the preferred transplantation protocol. The inconsistent improvement in hearing loss following transplantation suggests that HSCT may not be highly effective for this symptom [12–14]. In contrast, improvements in bony deformities and coarse facial features were observed after HSCT, with no reported joint abnormalities [12, 14–16, 18]. However, some patients did not experience these benefits [13, 17], likely due to variations in baseline disease severity [32, 33].

Despite its benefits, HSCT has significant risks of morbidity and mortality that are heavily influenced by the transplant protocol and donor match. In general, younger patients demonstrate a better risk‒benefit profile [10]. A follow-up study of 17 patients who underwent HSCT at a median age of 3.6 years reported 15 survivors after 5.5 years [13]. Serious and fatal pulmonary complications have also been documented post-HSCT in other metabolic disorders [13, 34]. Newer approaches such as PBSCT or pretransplant ERT combined with a matched unrelated cord blood transplant have shown no signs of chronic GVHD or other long-term complications [16, 18]. While these findings suggest a safer profile, they are based on small sample sizes, limiting their generalizability and highlighting the need for further research.

Since the European Medicines Agency authorized velmanase alfa (VA) in 2018 for long-term treatment of nonneurological symptoms in mild to moderate alpha-mannosidosis (AM) [35], several trials have evaluated its efficacy. Neurocognitive assessments in children have shown variable results, and no significant changes in CSF biomarkers have been detected [20–22]. These findings align with a posttrial study concluding that VA is not effective for improving cognitive function and is not recommended for neurological symptoms [36].

Hearing improvement varied, with bone conduction thresholds indicating inconsistent outcomes [20, 22]. However, VA had a positive impact on immune function, with some patients showing elevated IgA levels, supporting its role in immune system enhancement [20–21, 23].

Motor function assessments revealed improvements, particularly in patients who received longer treatment durations [20–22]. Pediatric patients demonstrated greater gains, notably in 3MSCT and 6MWT scores [20–21, 23], as well as respiratory function [21–23]. These findings highlight the importance of early ERT initiation, similar to HSCT. VA also led to reduced oligosaccharide levels in serum and CSF [20–23]. A long-term follow-up study recommended starting ERT even before clinical symptoms emerge, particularly in younger patients [37]. Although outcomes are more pronounced in children, adults can also benefit from ERT over time [38].

The quality of life improved following VA treatment [20]. A patient self-reported improvements in physical manifestations, such as joint pain, gait, and ear infections, after starting ERT at age 30 and continuing until age 34 [31]. Notably, the major advantage of VA over HSCT is its strong safety profile, with no reported mortality, making it suitable for long-term use [20–23].

Additionally, a study investigating anti-drug antibodies (ADAs) revealed that high ADA levels managed with corticosteroids, antihistamines, and slower infusion rates did not reduce the clinical effectiveness of VA. These findings suggest that VA remains safe and beneficial even in patients with high ADA levels [39].

A drawback of ERT is the requirement for lifelong weekly treatments and regular transportation to the hospital, which may add psychological and financial burdens to affected families [40].

Both HSCT and ERT provide considerable therapeutic advantages and improve patient outcomes when applied appropriately. The decision between HSCT and ERT depends on variables such as age at diagnosis, clinical severity, and treatment availability. The presence of neurocognitive involvement favors the use of HSCT, whereas the favorable safety profile of ERT makes it more suitable for managing nonneurological manifestations.

Early intervention with either option leads to better outcomes, emphasizing the importance of a timely diagnosis. This can be achieved by raising AM awareness among physicians and health care providers at the undergraduate and postgraduate levels and providing accessible and easily understandable information for parents online. Genetic testing can be used to identify asymptomatic siblings, support patient advocacy groups in sharing experiences, and reach a larger population [41].

As there are currently no established guidelines for managing AM, this study provides a comprehensive comparison of the two main therapies. It offers evidence-based insights to support clinical decision-making and improve patient care.

Our review is limited by the small sample sizes of many studies, which limits the generalizability of the findings. Additionally, the variations in study designs and patient populations contributes to the inconsistencies in the results.

## Conclusions

This review highlights the critical role of early intervention with HSCT and ERT in enhancing outcomes for individuals with AM. HSCT has shown promise in improving neurocognitive function and managing musculoskeletal and respiratory symptoms, especially when it is administered to pediatric patients. However, careful consideration of the benefit‒risk balance is crucial for mitigating serious morbidity and mortality concerns, particularly in older patients. On the other hand, ERT has a favorable safety profile and effectively improves respiratory function, musculoskeletal issues, and overall quality of life. Despite these advancements, challenges such as the lifelong commitment to ERT and the variability in treatment responses among different patient groups persist. Future research should prioritize large-scale, long-term studies to validate these findings, refine treatment protocols, and focus on identifying factors influencing therapeutic variability to tailor more personalized treatment strategies.

## Data Availability

Data sharing is not applicable to this article, as no datasets were generated or analyzed during the current study.

## Abbreviations

AM: Alpha-mannosidosis
HSCT: Hematopoietic stem cell transplantation
ERT: Enzyme replacement therapy
VA: Valemenase alfa
CENTRAL: Cochrane Central Register of Controlled Trials
PRISMA: Systematic reviews and meta-analyses
MeSH: Medical subject headings
RCTs: Randomized controlled trials
CNS: Central nervous system
Ig: Immunoglobulin
QoL: Quality of life
PBSCT: Peripheral blood stem cell transplant
MRI: Magnetic resonance imaging
CSF: Cerebrospinal fluid
GVHD: Graft-versus-host disease
3MSCT: 3-Minute Stair Climb Test
6MWT: 6-minute walk test
FVC: Forced vital capacity
ADA: Anti-drug antibodies
